# The potential dual effects of sevoflurane on AKT/GSK3β signaling pathway

**DOI:** 10.1186/2045-9912-4-5

**Published:** 2014-03-03

**Authors:** Lei Zhang, Jie Zhang, Yuanlin Dong, Celeste A Swain, Yiying Zhang, Zhongcong Xie

**Affiliations:** 1 Geriatric Anesthesia Research Unit, Department of Anesthesia, Critical Care and Pain Medicine, Massachusetts General Hospital and Harvard Medical School, 149 13th St., Room 4310, Charlestown, MA 02129-2060, USA; 2 Department of Anesthesiology, East Hospital, Tongji University School of Medicine, Shanghai 200120, P. R. China; 3 Research Center for Translational Medicine, East Hospital, Tongji University School of Medicine, Shanghai 200120, P. R. China; 4 Department of Anesthesiology, Tongji Hospital, Tongji Medical College, Huazhong University of Science and Technology, Wuhan, P.R. China

**Keywords:** Anesthetic, Sevoflurane, Phosphorylation, AKT/GSK3β signaling pathway

## Abstract

**Background:**

Anesthesia with multiple exposures of commonly used inhalation anesthetic sevoflurane induces neuroinflammation and cognitive impairment in young mice, but anesthesia with a single exposure to sevoflurane does not. AKT/glycogen synthase kinase 3β (GSK3β) signaling pathway is involved in neurotoxicity and neurobehavioral deficits. However, whether sevoflurane can induce a dual effect (increase versus decrease) on the activation of AKT/GSK3β signaling pathway remains to be determined. We therefore set out to assess the effects of sevoflurane on AKT/GSK3β signaling pathway *in vivo* and *in vitro*.

**Methods:**

Six day-old wild-type mice were exposed to 3% sevoflurane two hours daily for one or three days. In the *in vitro* studies, H4 human neuroglioma cells were treated with 4% sevoflurane for two or six hours. We then determined the effects of different sevoflurane treatments on the levels of phosphorylated (P)-GSK3β(ser9) and P-AKT(ser473) by using Western blot analysis.

**Results:**

Here we show that anesthesia with 3% sevoflurane two hours daily for one day increased the levels of P-GSK3β(ser9) and P-AKT(ser473), but the anesthesia with 3% sevoflurane daily for three days decreased them in the mice. The treatment with 4% sevoflurane for two hours increased, but the treatment with 4% sevoflurane for six hours decreased, the levels of P-GSK3β(ser9) and P-AKT(ser473) in the H4 human neuroglioma cells.

**Conclusions:**

Anesthetic sevoflurane might induce a dual effect (increase versus decrease) on the activation of the AKT/GSK3β signaling pathway. These studies have established a system to perform further studies to determine the effects of sevoflurane on brain function.

## Introduction

There are approximately six million children who undergo surgical care under anesthesia each year in America alone [1]. The increased use of anesthetics in children makes the safety of anesthesia a major health issue in the United States and in the world [[2], reviewed in [3]]. Several clinical studies have shown that anesthesia and surgery could be risk factors for subsequent cognitive impairment [reviewed in [3]]. Specifically, children may develop cognitive deficiency following multiple exposures (e.g., three times) to anesthesia and surgery at an early age (e.g., before age 4) [[4,5], reviewed in [3]]. These findings have become a major public health issue [2], and have promoted more clinical and pre-clinical investigations.

In the animal studies, it has been reported that anesthesia may induce neurotoxicity and neurobehavioral deficits in rodents [6-8] and monkeys [9,10] [reviewed in [3]]. A recent study has shown that anesthesia with 3% sevoflurane two hours daily for three, but not one, days induces neuroinflammation and cognitive impairment in young (six day-old) mice [11]. These studies suggested that sevoflurane might have dual effects on neurotoxicity and cognitive function.

Glycogen synthase kinase 3β (GSK3β) has been reported to contribute to Alzheimer’s disease neuropathogenesis, including β-amyloid protein and Tau [12], apoptosis, neuroinflammation, oxidative stress, acetylcholine activity, axon degeneration, and axonal transport, leading to cognitive impairment [[13]–[16], reviewed in [17]].

AKT is a serine/threonine kinase and is activated by phosphorylation under normal physiological conditions. The activated AKT [phosphorylated AKT (P-AKT)] then phosphorylates substrates, including GSK3β, to phosphorylated substrates, e.g., phosphorylated GSK3β (P-GSK3β) [18]. Specifically, AKT phosphorylates GSK3β at ser9 and the P-GSK3β (ser9), leading to decreased activity of GSK3β [19,20].

Activation of the AKT/GSK3β signaling pathway, demonstrated as increases in the levels of P-AKT(ser473) and P-GSK3β(ser9), has been reported to protect against myocardial apoptosis induced by ischemia/reperfusion in rats [21] and to increase the survival of hippocampal neurons following ischemia in rats [22,23].

We therefore set out to investigate the potential dual effects of sevoflurane anesthesia on the AKT/GSK3β signaling pathway in young (six day-old) wild-type mice and in H4 human neuroglioma cells (H4 cells). The hypothesis in the study is that single exposure or short duration treatment with sevoflurane increases, but multiple exposures or long duration treatment with sevoflurane decreases, the levels of P-GSK3β and P-AKT. We used 3% sevoflurane in the animal studies because our previous studies showed that anesthesia with 3% sevoflurane two hours daily for three days, but not one day, was able to induce neuroinflammation and cognitive impairment in mice [11]. We used 4% sevoflurane in the *in vitro* studies because our previous *in vitro* studies showed the treatment with 4% sevoflurane for six hours could induce apoptosis and increase Aβ levels in the human H4 neuroglioma cells [24].

## Materials and methods

### Mice anesthesia and treatment

All experiments were performed in accordance with the National Institute of Health guidelines and regulations. The animal protocol was approved by the Massachusetts General Hospital Standing Committee on the Use of Animals in Research and Teaching (Boston, Massachusetts). Efforts were made to minimize the number of animals used.

Both male and female mice (C57BL/6J, Jackson Lab, Bar Harbor, ME) were used in the studies. Young mice (six day-old) were used in the current studies. The mice were randomly assigned into the anesthesia group or the control group. The mice received the sevoflurane at postnatal day (P) 6 or from P6 to P8. The mice received anesthetic sevoflurane (3%) plus 60% oxygen (balanced with nitrogen) as performed in our previous studies [11,25]. The 60% oxygen maintains sufficient partial pressure of oxygen levels in the mice during anesthesia as demonstrated in previous studies [7,11,25]. The size of the induction chamber in the current study was 20 × 20 × 7 centimeters. The induction flow rate was 2 liters per minute for the first three minutes (for the induction) and then one liter per minute (for maintenance). Control group received 60% oxygen at an identical flow rate in similar chambers. The anesthetic and oxygen concentrations were measured continuously by a gas analyzer (Ohmeda, GE Healthcare, Tewksbury, MA). The temperature of the anesthetizing chamber was controlled by the DC Temperature Control System (FHC, Bowdoinham, Maine), which is a feedback based system for monitoring and controlling temperature, to maintain the rectal temperature of the mice as 37 ± 0.5°C. Previous studies [7,11,25] have shown that anesthesia with 3% sevoflurane for two hours did not significantly change the values of pH, partial pressure of oxygen, or partial pressure of carbon dioxide as compared to the control group. Mortality rate of mice in these studies was less than 1%.

### Cell lines and treatment

We employed H4 human neuroglioma cells (H4 cells) in the experiments. The cells were cultured in DMEM (high glucose) containing 9% heat-inactivated fetal calf serum, 100 units/ml penicillin, 100 μg/ml streptomycin, and 2 mM L-glutamine. The cells were treated with 21% O_2_, 5% CO_2_ and 4% [2 minimum alveolar concentration (MAC)] sevoflurane for two or six hours, as described by Dong et al. [24]. 21% O_2_, 5% CO_2_ and 4% sevoflurane were delivered from an anesthesia machine to a sealed plastic box in a 37°C incubator containing six-well plates seeded with one million cells in 1.5 ml cell culture media. A Datex infrared gas analyzer (Ohmeda, GE Healthcare) was used to continuously monitor the concentrations of delivered carbon dioxide, oxygen, and sevoflurane as performed in our previous studies [24].

### Harvest of brain tissues and cells, and protein level quantification

Following the anesthesia, the mice were killed by decapitation at P8. The brain tissues were harvested and subjected to Western blot analysis. The H4 cells were harvested in the end of the sevoflurane treatment or control condition. The harvested brain tissues and H4 cells were homogenized on ice using immunoprecipitation buffer (10 mM Tris–HCl, pH 7.4, 150 mM NaCl, 2 mM EDTA, 0.5% Nonidet P-40) plus protease inhibitors (1 μg/ml aprotinin, 1 μg/ml leupeptin, 1 μg/ml pepstatin A). The lysates were collected, centrifuged at 12,000 rpm for 10 minutes, and quantified for total proteins with bicinchoninic acid protein assay kit (Pierce, Iselin, NJ).

### Western blot analysis

GSK3β antibody (1:1,000 dilution, Cell Signaling Technology, #9336, Danvers, MA) was used to recognize P-GSK3β(ser9) (46 kDa). P-AKT(ser473) was recognized by P-AKT(ser473) antibody (60 kDa, 1:1,000, Cell Signaling Technology, #9271). Finally, the antibody to detect non-targeted protein β-Actin (42 kDa, 1:5,000, Sigma, St. Louis, MO) was used to control for loading differences in total protein amounts. Western blot quantification was performed as described by Zhang et al. [26]. Briefly, signal intensity was analyzed using image analysis program Quantity One (Bio-Rad, Hercules, CA). We quantified the Western blots in two steps, first using β-Actin levels to normalize protein levels (e.g., determining the ratio of P-GSK3β to β-Actin amount) and control for loading differences in the total protein amount. Second, we presented protein level changes in mice undergoing sevoflurane anesthesia as a percentage of those in the control condition. 100% of protein level changes refer to control levels for the purpose of comparison to experimental conditions.

### Statistics

Data were expressed as mean ± standard deviation (SD). Each group had 6 mice or wells of cells. We performed a power analysis based on our previous studies [11,24], and found that a sample size of 6 per arm would lead to a 90% power to detect a difference in the behavioral changes using a two-sided *t*-test with 5% type I error. Given the presence of background AKT/GSK3β activation in cells and brain tissues of mice, we did not use absolute values to describe these changes. Instead, these changes were presented as percentages of those from the control group. For example, one hundred percent of AKT refers to the control level for the purpose of comparison to experimental conditions. Student’s *t*-test was used to determine the difference between the sevoflurane anesthesia and control condition in the levels of P-GSK3β and P-AKT. P values less than 0.05 were considered statistically significant. Prism 6 software (La Jolla, CA) was used to analyze the data.

## Results

### Single exposure with sevoflurane anesthesia in young WT mice increased the levels of P-GSK3β(ser9) and P-AKT(ser473) in brain tissues of the mice

Activation of AKT/GSK3β signaling pathway, demonstrated as increases in the levels of P-GSK3β(ser9) and P-AKT(ser473), has been reported to protect cellular toxicity [21-23]. We therefore set out to study the effects of sevoflurane anesthesia on the levels of P-GSK3β(ser9) and P-AKT(ser473).

**Figure 1:**
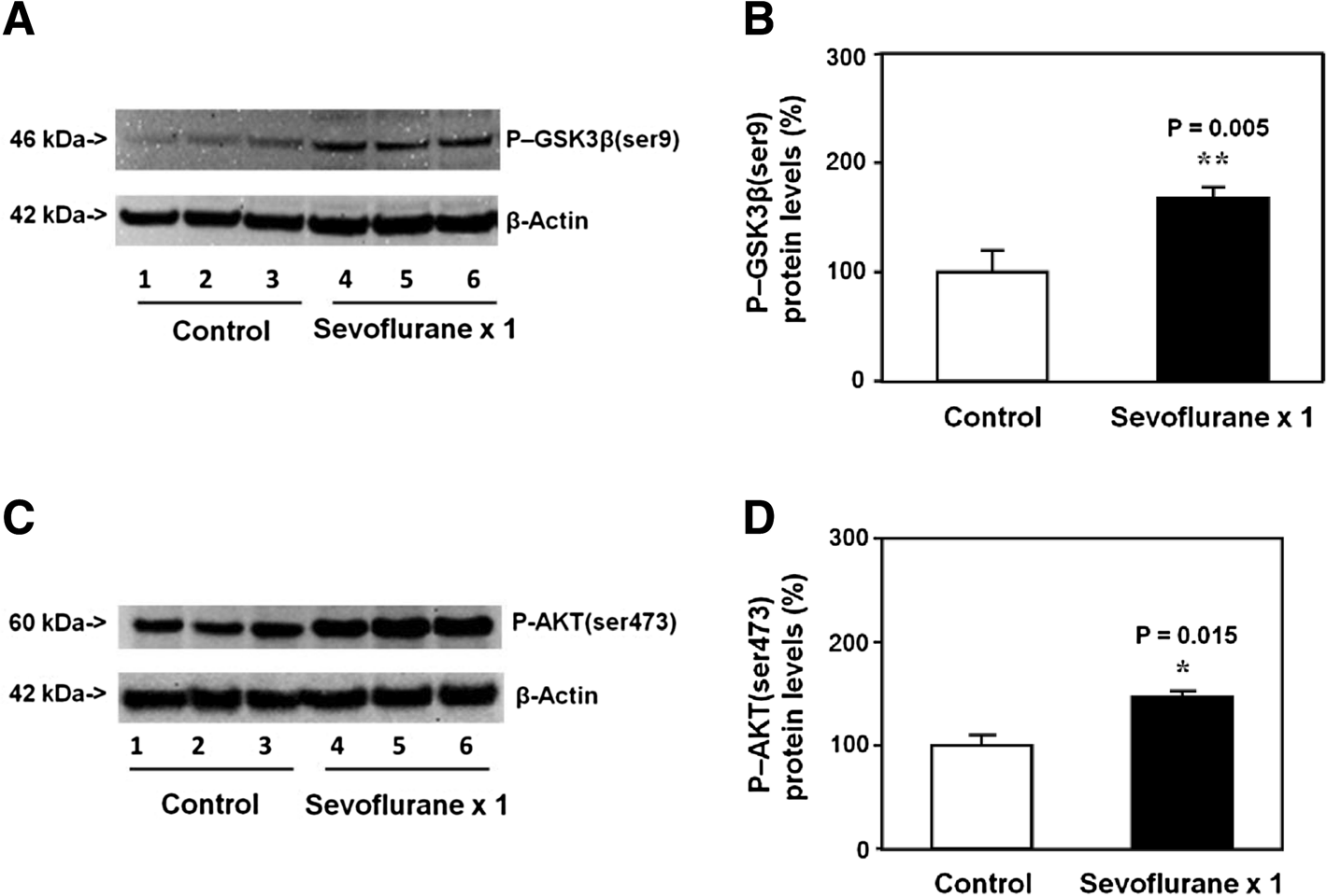
**Single sevoflurane anesthesia in P6 mice increases levels of P-GSK3β(ser9) and P-AKT(ser473) in brain tissues. A**. Anesthesia with 3% sevoflurane two hours daily for one day in P6 mice increases the levels of P-GSK3β(ser9) in the brain tissues of the mice as compared to the control condition. There is no significant difference in β-Actin levels in the brain tissues of the mice between the sevoflurane anesthesia and control condition. **B**. Quantification of the Western blot shows that the sevoflurane anesthesia increases the levels of P-GSK3β(ser9) in the brain tissues of the mice as compared to the control condition. **C**. Anesthesia with 3% sevoflurane two hours daily for one day in P6 mice increases the levels of P-AKT(ser473) in the brain tissues of the mice as compared to the control condition. There is no significant difference in β-Actin levels in the brain tissues of the mice between the sevoflurane anesthesia and control condition. **D**. Quantification of the Western blot shows that the sevoflurane anesthesia increases the levels of P-AKT(ser473) in the brain tissues of the mice as compared to the control condition. P, phosphorylated; GSK3β, glycogen synthase kinase 3β. N = 6.

P-GSK3β(ser9) immunoblotting showed that there was a visible increase in the levels of P-GSK3β(ser9) in brain tissues of the mice treated with sevoflurane (lanes 4 to 6) as compared to that of the mice treated with the control condition (lanes 1 to 3) (Figure 1A). There was no significant difference in the β-Actin levels in the brain tissues of the mice treated with sevoflurane as compared to that of the mice treated with the control condition (Figure 1A). Quantification of the Western blot, based on the ratio of P-GSK3β(ser9) levels to β-Actin levels, showed that the sevoflurane anesthesia (black bar) increased the P-GSK3β(ser9) levels as compared to the control condition (white bar): 182% versus 100%, P = 0.005 (Student *t*-test) (Figure 1B).

Next, we assessed the effects of the sevoflurane anesthesia on P-AKT(ser473) levels in the brain tissues of mice. P-AKT(ser473) immunoblotting showed that there was a visible increase in the levels of P-AKT(ser473) in brain tissues of the mice treated with sevoflurane (lanes 4 to 6) as compared to that of the mice treated with the control condition (lanes 1 to 3) (Figure 1C). There was no significant difference in the β-Actin levels between the sevoflurane anesthesia and the control condition (Figure 1C). Quantification of the Western blot, based on the ratio of P-AKT(ser473) levels to β-Actin levels, showed that the sevoflurane anesthesia (black bar) increased the P-AKT(ser473) levels as compared to control condition (white bar): 153% versus 100%, P = 0.015 (Student *t*-test) (Figure 1D).

### Multiple exposures with sevoflurane anesthesia in young WT mice decreased the levels of P-GSK3β(ser9) and P-AKT(ser473) in the brain tissues of the mice

Our previous studies have shown that anesthesia with 3% sevoflurane two hours daily for one day in P6 does not induce cognitive impairment in the mice at P30. However, the anesthesia with 3% sevoflurane two hours daily for three days (from P6 to P8) is able to induce cognitive impairment in the mice at P30 [11]. Given that single sevoflurane anesthesia increased the levels of P-GSK3β(ser9) and P-AKT(ser473), next, we assessed the effects of multiple exposures with sevoflurane anesthesia on the levels of P-GSK3β(ser9) and P-AKT(ser473) in the brain tissues of young mice.

**Figure 2:**
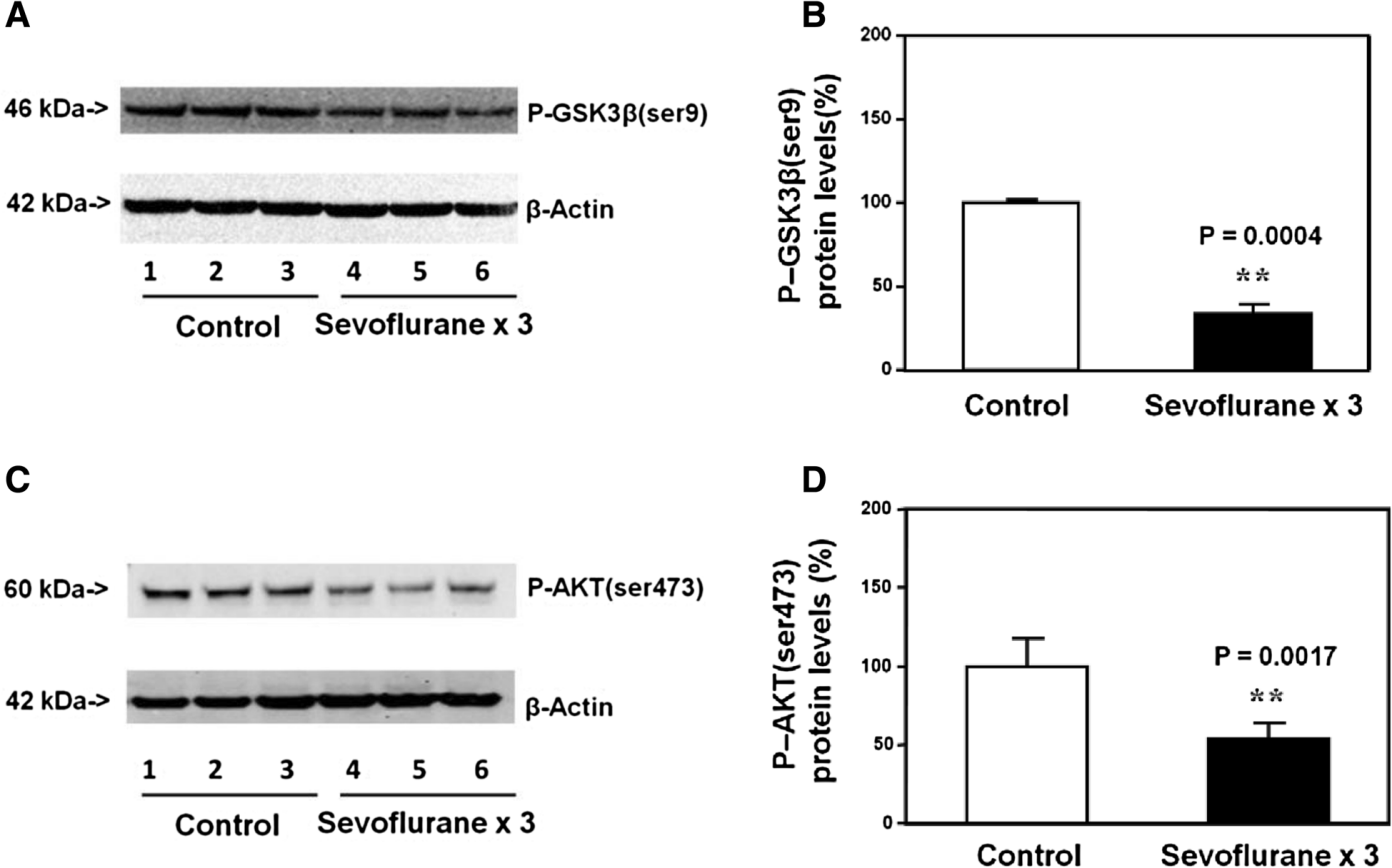
**Multiple sevoflurane anesthesia in P6 mice decreases levels of P-GSK3β(ser9) and P-AKT(ser473) in brain tissues. A**. Anesthesia with 3% sevoflurane two hours daily for three days in P6 mice decreases the levels of P-GSK3β(ser9) in the brain tissues of the mice as compared to the control condition. There is no significant difference in β-Actin levels in the brain tissues of the mice between the sevoflurane anesthesia and control condition. **B**. Quantification of the Western blot shows that the sevoflurane anesthesia decreases the levels of P-GSK3β(ser9) in the brain tissues of the mice as compared to the control condition. **C**. Anesthesia with 3% sevoflurane two hours daily for three days in P6 mice decreases the levels of P-AKT(ser473) in the brain tissues of the mice as compared to the control condition. There is no significant difference in β-Actin levels in the brain tissues of the mice between the sevoflurane anesthesia and control condition. **D**. Quantification of the Western blot shows that the sevoflurane anesthesia decreases the levels of P-AKT(ser473) in the brain tissues of the mice as compared to the control condition. P, phosphorylated; GSK3β, glycogen synthase kinase 3β. N = 6.

P-GSK3β(ser9) immunoblotting demonstrated a visible decrease in the levels of P-GSK3β(ser9) in brain tissues of the mice treated with the sevoflurane anesthesia (lanes 4 to 6) as compared to that of the mice treated with control condition (lanes 1 to 3) (Figure 2A). There was no significant difference in the β-Actin levels between the sevoflurane anesthesia and the control condition (Figure 2A). Quantification of the Western blot, based on the ratio of P-GSK3β(ser9) levels to β-Actin levels, indicated that the sevoflurane anesthesia (black bar) decreased the P-GSK3β(ser9) levels as compared to control condition (white bar): 37% versus 100%, P = 0.0004 (Student *t*-test) (Figure 2B).

The Western blot analysis also showed that the anesthesia with 3% sevoflurane two hours daily for three days (lanes 4 to 6) decreased the levels of P-AKT(ser473) as compared to the control condition (lanes 1 to 3) in the brain tissues of the mice. There was no significant difference in the β-Actin levels in the brain tissues between the sevoflurane anesthesia and control condition. The quantification of the Western blot demonstrated that the sevoflurane anesthesia (black bar) decreased the levels of P-AKT(ser473): 53% versus 100%, P = 0.0017 (Student *t*-test) (Figure 2D).

These findings suggested that the single exposure with 3% sevoflurane anesthesia for two hours increased the levels of P-GSK3β(ser9) and P-AKT(ser473) as compared to the control condition, but the multiple (three times) exposures with 3% sevoflurane anesthesia for two hours decreased the levels of P-GSK3β(ser9) and P-AKT(ser473), as compared to the control condition in the brain tissues of young mice.

### Short time treatment with sevoflurane in H4 cells increased the levels of P-GSK3β(ser9) and P-AKT(ser473) in the H4 cells

Given the findings that there was a difference in the levels of P-GSK3β(ser9) and P-AKT(ser473) between single (two hours) and multiple (three two hour) exposures of anesthesia with sevoflurane in the brain tissues of mice, next, we asked whether such difference was owing to multiple exposures to sevoflurane or was also owing to the longer duration of anesthesia. It is difficult to anesthetize young mice with 3% sevoflurane for six hours because such anesthesia was reported to induce high mortality rate [25]. Therefore, we assessed the potential different effects between two hours and six hours anesthesia with sevoflurane on the levels of P-GSK3β(ser9) and P-AKT(ser473) in H4 cells.

**Figure 3:**
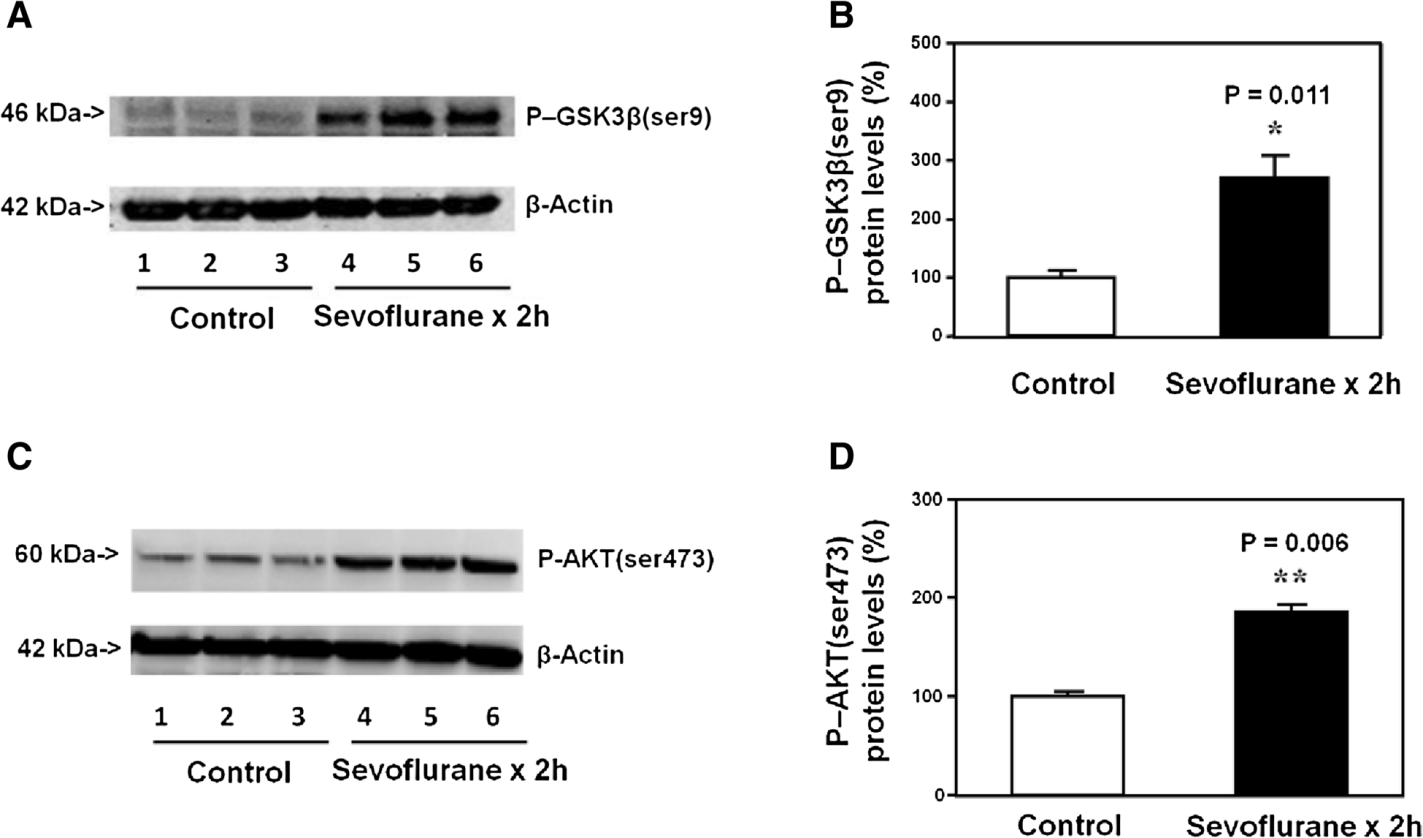
**Short sevoflurane treatment increases the levels of P-GSK3β(ser9) and P-AKT(ser473) in H4 cells. A**. Anesthesia with 4% sevoflurane for two hours increases the levels of P-GSK3β(ser9) in the H4 cells as compared to the control condition. There is no significant difference in β-Actin levels in the H4 cells between the sevoflurane anesthesia and control condition. **B**. Quantification of the Western blot shows that the sevoflurane anesthesia increases the levels of P-GSK3β(ser9) in the H4 cells as compared to the control condition. **C**. Anesthesia with 4% sevoflurane for two hours in H4 cells increases the levels of P-AKT(ser473) in the H4 cells as compared to the control condition. There is no significant difference in β-Actin levels in the H4 cells between the sevoflurane anesthesia and control condition. **D**. Quantification of the Western blot shows that the sevoflurane anesthesia increases the levels of P-AKT(ser473) in the H4 cells as compared to the control condition. P, phosphorylated; GSK3β, glycogen synthase kinase 3β. N = 6.

P-GSK3β(ser9) immunoblotting showed a visible increase in the levels of P-GSK3β(ser9) in the H4 cells treated with 4% sevoflurane for two hours (lanes 4 to 6) as compared to that of the cells treated with the control condition (lanes 1 to 3) (Figure 3A). There was no significant difference in the β-Actin levels in the H4 cells treated with sevoflurane as compared to that of the H4 cells treated with the control condition (Figure 3A). Quantification of the Western blot, based on the ratio of P-GSK3β(ser9) levels to β-Actin levels, showed that the sevoflurane treatment (black bar) increased the P-GSK3β(ser9) levels as compared to the control condition (white bar): 277% versus 100%, P = 0.011 (Figure 3B) (Student *t*-test).

The Western blot analysis showed that the treatment with 4% sevoflurane for two hours (lanes 4 to 6) also increased P-AKT(ser473) levels as compared to the control condition (lanes 1 to 3) in the H4 cells (Figure 3C). There was no significant difference in the β-Actin levels between the sevoflurane treatment and the control condition (Figure 3C). Quantification of the Western blot, based on the ratio of P-AKT(ser473) levels to β-Actin levels, showed that the sevoflurane treatment (black bar) increased the P-AKT(ser473) levels as compared to the control condition (white bar) in the H4 cells: 188% versus 100%, P = 0.006 (Student *t*-test) (Figure 3D).

### Long time treatment with sevoflurane in H4 cells decreased the levels of P-GSK3β(ser9) and P-AKT(ser473) in the H4 cells

**Figure 4:**
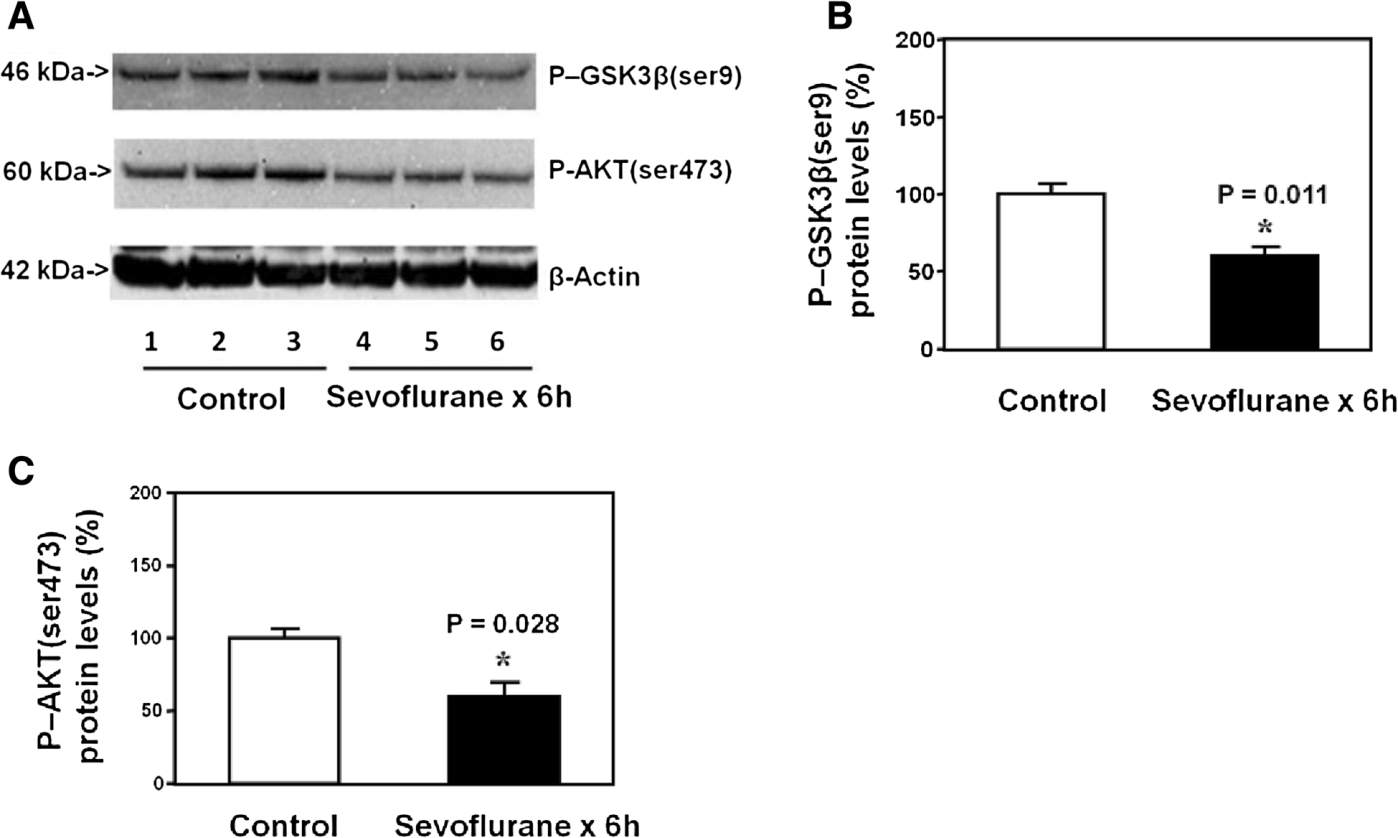
**Long sevoflurane treatment decreases the levels of P-GSK3β(ser9) and P-AKT(ser473) in H4 cells. A**. Anesthesia with 4% sevoflurane for six hours decreases the levels of P-GSK3β(ser9) and P-AKT(ser473) in the H4 cells as compared to the control condition. There is no significant difference in β-Actin levels in the H4 cells between the sevoflurane anesthesia and control condition. **B**. Quantification of the Western blot shows that the sevoflurane anesthesia decreases the levels of P-GSK3β(ser9) in the H4 cells as compared to the control condition. **C**. Quantification of the Western blot shows that the sevoflurane anesthesia decreases the levels of P-AKT(ser473) in the H4 cells as compared to the control condition. N = 6.

Finally, we asked whether long time treatment with sevoflurane might have different effects on the levels of P-GSK3β(ser9) and P-AKT(ser473) in the H4 cells. The Western blot analysis showed that the treatment with 4% sevoflurane for six hours (lanes 4 to 6) reduced the levels of both P-GSK3β(ser9) and P-AKT(ser473) as compared to the control condition (lanes 1 to 3) in the H4 cells (Figure 4A). There was no significant difference in the β-Actin levels between the sevoflurane treatment and the control condition (Figure 4A). The quantification of the Western blot, based on the ratio of the P-GSK3β(ser9) and P-AKT(ser473) levels to the β-Actin levels, showed that the sevoflurane treatment (4% and six hours, black bar) decreased the levels of P-GSK3β(ser9) (Figure 4B, 57% versus 100%, P = 0.011, Student *t*-test) and P-AKT(ser473) (Figure 4C, 52% versus 100%, P = 0.028, Student *t*-test) as compared to the control condition (white bar).

Taken together, these data suggested that different durations (two versus six hours) of sevoflurane treatment in H4 cells and different exposures of sevoflurane anesthesia (one versus three times) in mice could lead to varying effects (increase versus reduction) on the levels of P-GSK3β(ser9) and P-AKT(ser473).

## Discussion

Sevoflurane is the most commonly used anesthetic in children. Sevoflurane has been shown to induce apoptosis [7,25], increase β-amyloid protein levels [25] and neuroinflammation [11], leading to cognitive impairment in young mice [7,11,25]. Specifically, anesthesia with 3% sevoflurane two hours daily for three days induces neuroinflammation and cognitive impairment, while anesthesia with 3% sevoflurane two hours daily for one day does not [11]. These data suggested that anesthetic sevoflurane might cause dual effects on neurotoxicity and cognitive function. However, whether sevoflurane anesthesia can induce dual effects on AKT/GSK3β signaling pathway remains to be determined.

We first found that anesthesia with 3% sevoflurane two hours daily for one day increased the levels of P-GSK3β(ser9) and P-AKT(ser473) (Figure 1). These data indicated that the single exposure to sevoflurane might enhance the activation of the AKT/GSK3β signaling pathway. However, the anesthesia with 3% sevoflurane two hours daily for three days reduced the levels of P-GSK3β(ser9) and P-AKT(ser473) (Figure 2). These results showed that the multiple exposures to sevoflurane decreased the activation of the AKT/GSK3β signaling pathway. These findings suggested that single (two hours) and multiple (three times of two hours) exposures with sevoflurane anesthesia could increase and decrease the activation of AKT/GSK3β signaling pathway, respectively, potential dual effects of sevoflurane on the AKT/GSK3β signaling pathway. The future studies might include a different anesthesia regimen to further test this hypothesis. We would assess whether anesthesia with 3% sevoflurane two hours weekly for one or three weeks might have different effects on the AKT/GSK3β signaling pathway as well as other behavioral and brain biochemistry changes.

Such a difference could result from single versus multiple exposures to sevoflurane or from shorter versus longer durations of anesthesia. Anesthetizing young mice with 3% sevoflurane for six hours could lead to a high mortality rate [25]. Therefore, H4 cells were used to assess the potential difference of short versus long duration of sevoflurane anesthesia on the levels of P-GSK3β(ser9) and P-AKT(ser473).

We were able to show that the treatment with 4% sevoflurane for two hours increased, but the treatment with 4% sevoflurane for six hours decreased, the levels of P-GSK3β(ser9) and P-AKT(ser473). These data showed the potential dual effects of sevoflurane in H4 cells and that a short duration of sevoflurane anesthesia increased the activation of the AKT/GSK3β signaling pathway, but a long duration of sevoflurane anesthesia decreased it.

Phosphorylation at serine 9 of GSK3β inhibits the activity of GSK3β [27-32]. The current findings that the short exposure time to sevoflurane anesthesia increased P-GSK3β(ser9) levels, but the longer exposure to sevoflurane anesthesia exposure decreased P-GSK3β(ser9) levels, indicates that the short exposure time to sevoflurane anesthesia reduced, but the longer exposure time to sevoflurane enhanced the activity of GSK3β, respectively.

Taken together, these findings suggested that the dual effects of sevoflurane on AKT/GSK3β signaling pathway and that a short exposure time to sevoflurane treatment might produce neuroprotection via activation of AKT/GSK3β signaling pathway, but a long exposure time to sevoflurane anesthesia could induce neurotoxicity via inhibition of AKT/GSK3β signaling pathway. Interestingly, while long durations (e.g., three time of two hours) of sevoflurane anesthesia induced cognitive impairment, the shorter exposure time (e.g., one time of two hours) of sevoflurane anesthesia did not improve the learning and memory function in the mice [11]. It is conceivable that the short exposure time to sevoflurane anesthesia may only produce neuroprotection when there is a brain insult. This hypothesis has been supported by the outcomes from the previous studies which show that sevoflurane has neuroprotective effects [reviews in [33,34]]. The findings that sevoflurane may have dual effects (neuroprotection versus neurotoxicity) would be important to further determine the role of sevoflurane in brain function. In addition, our studies showed that the AKT/GSK3β could be one of the cellular mechanisms by which sevoflurane produced the dual effects. These results suggested that regulation of AKT/GSK3β by anesthetics or other perioperative factors might affect brain function during surgery. The future studies to assess whether short exposure time to sevoflurane (or other anesthetics) anesthesia attenuates, but long durations of sevoflurane anesthesia potentiates, brain insults, e.g., cerebral ischemia, as well as studies to understand the underlying mechanisms are needed.

The current studies have several limitations. First, we did not determine the ratio of P-GSK3β(ser9) to total GSK3β and the ratio of P-AKT(ser473) to total AKT. However, the changes in the levels of P-GSK3β(ser9) and P-AKT(ser473) are sufficient to reflect the changes in the AKT/GSK3β signaling pathway [21-23]. Second, we did not assess the downstream outcomes of the AKT/GSK3β signaling pathway following the different sevoflurane anesthesia. Such outcomes include many cellular changes, and may take a long time to complete. Third, different treatments with sevoflurane (e.g., once every three days) and different time intervals of brain harvest (three days after the sevoflurane anesthesia) may have different findings in regards to the potential dual effects of sevoflurane on brain function. However, such studies may exceed the scope of the current experiments, which aimed to establish a system and to generate a hypothesis for future studies. Nevertheless, the current experiments have established the system and demonstrated the effects of different sevoflurane anesthesia on the activation of the AKT/GSK3β signaling pathway. The future studies will employ the established system to systematically determine the dual effects of anesthetic sevoflurane on the AKT/GSK3β signaling pathway, including the time course studies, and investigation of the up-stream regulators and down-stream consequences.

## Conclusion

In conclusion, we found that anesthesia with 3% sevoflurane for two hours daily for one day increased the levels of P-GSK3β(ser9) and P-AKT(ser473) in the brain tissues of young mice, but the anesthesia with 3% sevoflurane for two hours daily for three days decreased the levels. Similarly, the anesthesia with 4% sevoflurane for two hours increased the levels of P-GSK3β(ser9) and P-AKT(ser473) in the H4 cells, but the anesthesia with 4% sevoflurane for six hours decreased them. These results have suggested the dual effects of sevoflurane; that a short duration of sevoflurane anesthesia activates, but a long exposure to sevoflurane anesthesia inhibits, the AKT/GSK3β signaling pathway. These findings have established a system and have shown the potential dual effects of sevoflurane anesthesia on the AKT/GSK3β signaling pathway, which will likely promote more studies to investigate the effects of sevoflurane on brain function.

## Abbreviations

SD: Standard deviation; GSK3β: Glycogen synthase kinase 3β; P-AKT: Phosphorylated AKT; P-GSK3β: Phosphorylated glycogen synthase kinase 3β.

## Competing interests

The authors have no conflicts of interest for the study.

## Authors’ contributions

LZ Study concept and design, Acquisition of data, Analysis and interpretation of data. JZ, YZ, and YD Analysis and interpretation of data, Drafting of the manuscript, Critical revision of the manuscript for important intellectual content. ZX Obtained funding, Study concept and design, Analysis and interpretation of data, Drafting of the manuscript, Critical revision of the manuscript for important intellectual content, Study supervision. All authors read and have approved the manuscript.
